# Work engagement and its association with occupational sitting time: results from the Stormont study

**DOI:** 10.1186/s12889-015-1427-9

**Published:** 2015-01-29

**Authors:** Fehmidah Munir, Jonathan Houdmont, Stacy Clemes, Kelly Wilson, Robert Kerr, Ken Addley

**Affiliations:** School of Sport, Exercise and Health Sciences, National Centre for Sport and Exercise Medicine, Loughborough University, Loughborough, Leicestershire LE11 3TU UK; School of Medicine, University of Nottingham, Yang Fujia Building, Jubilee Campus, Wollaton Road, Nottingham, NG8 1BB UK; Department of Management and Leadership, University of Ulster, Jordanstown Campus, Shore Road, Newtownabbey, Co. Antrim BT37 OQB UK; Northern Ireland Civil Service Occupational Health Service, Lincoln Building, 27-45 Great Victoria Street, Belfast, BT2 7SH UK

**Keywords:** Sedentary lifestyle, Work engagement, Employees, Occupational health, Health behavior

## Abstract

**Background:**

Evidence suggests that poor health outcomes and poor work-related health outcomes such as sickness presenteeism are associated with excessive sitting at work. Studies have yet to investigate the relationship between work engagement and occupational sitting. Work engagement is considered to be an important predictor of work-related well-being. We investigated the relationship between and self-reported work engagement and high occupational sitting time in Northern Ireland Civil Service (NICS) office-based workers.

**Method:**

A cohort of 4436 NICS office-workers (1945 men and 2491 women) completed a questionnaire measuring work engagement and occupational sitting time. Logistic regression analyses were used to test the associations between work engagement and occupational sitting times.

**Results:**

Compared to women, men reported lower mean occupational sitting time (385.7 minutes/day; s.d. = 1.9; versus 362.4 minutes/day; s.d. =2.5; p < .0001). After adjusting for confounding variables, men with high work engagement of vigor (OR = 0.49, 95% CI 0.34-0.98) and dedication (OR 0.68 95% CI 0.47-0.98) were less likely to have prolonged sitting time. Women with high work engagement of vigor (OR = 0.62, 95% CI 0.45-0.84) were also less likely to have prolonged occupational sitting times. In contrast, women with high absorption (OR = 1.29, 95% CI 1.01-1.65) were more likely to have prolonged sitting times.

**Conclusions:**

Being actively engaged in one’s work is associated with lower occupational sitting times for men (vigor and dedication) and to a limited extent for women (vigor only). This suggests that interventions such as introducing sit-stand workstations to reduce sitting times, may be beneficial for work engagement.

## Background

Healthy workers are important for the labour market, especially in today’s society of an ageing workforce and increasing prevalence of chronic diseases [1,2]. Workers who have poor health are more likely to have high sickness absence, exit early from the labour market or take early retirement [3]. One major risk factor to workers’ health is sedentary behaviour. Sedentary behaviour is defined as “any waking behaviour characterised by an energy expenditure of ≤1.5 metabolic equivalents (METs) while in a sitting or reclining posture” (page 540) [4,5]. Working adults spend around 70-80% of their working time sedentary [6]. Evidence suggest that sedentary behaviour is associated with poor health outcomes including obesity [6,7], type 2 diabetes [8], the metabolic syndrome [9], some cancers [10-12], and mortality from all-causes and cardiovascular disease [12,13]. Specifically, sedentary behaviour at work has been associated with both poor health outcomes [14] and work-related health outcomes such as sickness presenteeism (a reduced ability to work productively due to physical or psychological health conditions) [15]. Other studies have found an association between work stress and sedentary lifestyle and between low job control and physical inactivity [16,17].

The problems of prolonged sitting at work have led to recent workplace intervention studies to reduce sitting behaviour and promote standing and light movement [18]. Although these interventions reduce occupational sitting time and enhance health and wellbeing with no adverse effect on work performance as assessed for example, by number of words typed on a keyboard [19-21], studies have yet to investigate the relationship between occupational sitting and work engagement. Work engagement is considered to be an important predictor of work-related well-being [22]. It is characterised by high levels of personal energy, mental resilience and persistence in the face of difficulties (vigor and vitality), a sense of work significance, inspiration and enthusiasm (dedication); and being happily immersed in one’s work (absorption) [23]. Work engagement is positively related to work performance [22], and positively related to job resources (the physical, psychological, social, or organizational resources an individual has to deal with job demands such as having control over one’s job) [24]; and is negatively associated with sickness absenteeism [22] burnout and high job demands [25]. Many business organisations worldwide collect employee survey data on work engagement as a key indicator for employee health, well-being and productivity [26].

To date, research on occupational sitting has not examined the potential effect of occupational sitting time on work engagement. As work engagement is an active, positive state, it is possible that employees who have high levels of personal energy and inspiration are also likely to sit less by standing up and stretching their legs and/or taking regular short breaks to engage in activities such as talking to other colleagues about work tasks. This break from work in turn, may provide an opportunity to aid recovery and enhance personal resources such as replenish energy levels and arousal [27]. A recent study found that levels of psychological arousal increased with the use of active workstations [28]. It also found that use of active workstations (which provided employees a brief break from work by standing up, adjusting height of desk and then returning to work tasks whilst standing or walking on a treadmill) increased work task satisfaction and reduced levels of boredom and stress. We therefore hypothesize that there will be an association between work engagement and occupational sitting time; as well as between job performance and occupational sitting and between job strain (defined by a ratio of job demands to job control) [29] and occupational sitting, independent of individual health indices (such as BMI) in a large sample of office workers.

Whilst no systematic differences between gender have been reported in the literature for work engagement, gender differences have been observed in sedentary behaviours between men and women with men reporting significantly higher occupational sitting time than women in some studies [e.g. 6], and other studies reporting women with higher occupational sitting time [e.g. 7]. It was therefore hypothesized that there would be a mean difference in occupational sitting time between men and women. As there is insufficient knowledge as to why there are differences in occupational sitting time between men and women office workers and what factors might influence these, we examined the associations between the variables separately for men and women.

## Methods

This is a new study tracking a large cohort of public sector employees through and beyond their career with the Northern Ireland Civil Service (NICS). It includes staff working for the 12 Northern Ireland ministerial departments including the Public Prosecution Service for Northern Ireland. The NICS employed 27,739 employees in 2012 of which approximately 26,000 had access to email and were sent a survey link. 5235 employees (20% response rate) completed the questionnaire. For this study, we excluded questionnaires completed by manual workers and non-permanent staff (n = 641) and those office-based workers with missing data (n = 158). This resulted in 4436 questionnaires that were used in subsequent analyses. Table 1 shows participants’ demographic and occupational characteristics compared to the overall NICS employee profile. No significant differences were evident in terms of working hours. Differences were observed for gender, age and job grade but this may reflect non-permanent and manual workers in the NICS data. The majority of office-based jobs in NICS are classified as highly sedentary involving mostly desk work with two 15 minute coffee break and one 30 minute lunch break over a standard 8 hour day (with some variations across different sites). A Senior Civil Servant role includes mainly managerial/administrative tasks such as dealing with policy, strategy, supporting Ministers in the Assembly. Principal and Senior Principal are responsible for policy, service and operational delivery and staff management; and tend to head various branches within Divisions; Deputy Principal and Staff Officer provide support to Principal and Senior Principal staff . Executive Officers are largely in a direct line management role for the clerical staff. Administrative Officer/Assistant represents the largest group within the NICS. They carry out a range of administrative tasks including filing, answering telephones, photocopying etc. They have no line management role.

**Table 1:** **Comparison of respondents’ (n = 4436) demographic and occupational characteristics against Northern Ireland civil service population**

	**Survey respondents** **(October 2012)** ***n*** **%**	**Total NICS staff (April 2012)** ***n*** **%**	**Chi-square**	**P value**
**Gender**				
Male	1,945 (43.8)	12, 598 (45.8)	5.878	0.015
Female	2,491 (56.2)	14, 909 (54.2)		
**Age**				
≤24	67 (1.2)	655 (2.4)	295.847	0.0001
25-34	1,859 (34.2)	6,517 (23.5)		
35-44	1,153 (21.2)	7,198 (25.9)		
45-54	1,676 (30.8)	9,506 (34.3)		
≥55	681 (12.5)	3,863 (13.9)		
**Job grade** (in descending order)				
≥Senior civil servant/principal	389 (8.8)	1627 (6.1)	724.043	0.0001
Deputy principal	554 (15.0)	2,382 (8.9)		
Staff officer	898 (20.2)	3,288 (12.3)		
Executive officer	1,403 (31.6)	7,828 (29.3)		
Administrative	1,005 (22.7)	10,037 (37.6)		
Other	77 (1.7)	1,520 (5.7)		
**Working hours**				
Full time (≥ 37.5 hours per week)	3,672 (82.8)	22,654 (81.7)	3.162	0.075
Part time, job share, term time (less than 37.5 hours per week)	764 (17.2)	5,085 (18.3)		

The research was commissioned by the NICS Workplace Health Committee and ethical approval granted by the Ethics Committee of the University of Ulster.

### Occupational sitting

Occupational sitting time was measured with the Domain-Specific Sitting Time Questionnaire [30]. This self-report tool is a valid and reliable measure of sitting time in adults [30,31] which asks how much time respondents spend sitting (in hours and minutes) at work on a typical workday.

### Self-report physical activity

Physical activity was assessed by a valid and reliable single-item question asking participants to indicate how many days in the last 7 days they have undertaken 30 minutes or more of physical activity which was enough to raise their breathing rate [32]. This measure has been shown to offer a valid and reliable assessment of physical activity [33,34]. Participants were classified as meeting the UK government’s 2004 Physical activity guidelines if they reported participating in at least 30 minutes of moderate-to-vigorous physical activity on ≥5 days (yes/no).

### Work engagement

The nine-item Utrecht Work Engagement Scale was used to assess work engagement [18]. This frequently used scale captures ‘how workers experience their work’ [35] and is related to work productivity and general well-being [36,37]. The questionnaire has three subscales on *vigor,* defined as bringing in personal energy and resilience, willingness to invest effort, persisting with difficulties (example item: “At my work, I feel bursting with energy”), *dedication,* defined as deriving a sense of significance from one’s work (example item: “My job inspires me”), and *absorption*, defined as being totally and happily immersed in one’s work and having difficulties detaching oneself from it so that time passes quickly (e.g. “I feel happy when I am working intensely”). Responses are measured on a seven-point Likert scale from 0 = Never to 6 = Always. A mean score is calculated for each subscale. This scale has high factorial validity, internal consistency and stability [23]. In the current study, the alpha co-efficient for the scale was 0.90, and therefore considered to have internal reliability.

### Job demands and job control

The Management Standards Indicator tool (MSIT; Health and Safety Executive, no date available) is designed to aid organizations in meeting their legal duty for psychosocial risk assessment and is also used by researchers. Two subscales were used from the 35-item self-report Management Standards Indicator Tool (job demands (8 items) and job control (6 items). Responses are measured on a five-point scale from 1 = never, to 5 = always. Internal consistency is high across both scales and across all samples. Information on the tool can be found from the Health and Safety Executive website (http://www.hse.gov.uk/stress/standards/downloads.htm). Each scale is summed. Low scores are indicative of high exposure to psychosocial hazards. For logistic regression analyses, we calculated ratios for high job strain and low job strain (see analysis section).

### Job performance

Four items measured job performance [38] on a seven-point Likert Scale : ‘I perform the tasks that are expected of me’, ‘I go out of my way to help colleagues’, ‘I take time to take a personal interest in other employees’, and ‘I assist my supervisor/manager with his/her work even when not asked’. These items have good reported inter-correlations and reliability [38]. In the current study the alpha co-efficient for the scale was 0.77. A mean score is calculated.

### Covariates

Other measures included age (years), sex, smoking (yes/no) height (metres or feet and inches) and weight (kilograms) for BMI calculation purposes (kg/m^2^), marital status (single/married or cohabiting), dependents (yes/no), educational level, working hours (part time i.e. less than 35.5 hours /full-time i.e. ≥37.5 hours) and job grade (categories supplied by NICS; due to small Ns in ‘Senior Civil Servant’ Category, this was combined with the next Category ‘Senior Principal’). Using BMI, participants were categorized as normal weight (BMI <25 kg/m^2;^ this category included nine cases of underweight (BMI under 18.5), as there were too few cases to warrant a fourth category), overweight (BMI 25–29.9 kg/m^2^) or obese (BMI ≥30 kg/m^2^).

#### Analysis

Spearman correlations were carried out to examine the associations between individual characteristics and measures of work engagement and job performance. A *t*-test was conducted to examine the differences in mean sitting time between men and women. Associations between categorical variables were tested using chi-square analyses. Logistic regressions were performed to estimate the association between work engagement, job strain and work performance and the likelihood of moderate to high occupational sitting (against the reference category of low occupational sitting), as expressed by odds ratio (OR) and 95% confidence interval (CI). First, using a similar method reported in other studies [6,7] occupational sitting time was categorised into tertiles of low (<360 minutes/day) moderate (360-420 minutes/day), and high sitting time (421-840 minutes/day). Separate constructs of work engagement were categorised into tertiles of low, medium and high work engagement [18]. For absorption, there were no participant data for the low absorption category. Therefore only two categories were used. For job demands, ratios were calculated for high strain (where participants report high job demands and low job control – high strain is defined as negative psychological and physical strain) and low strain (where participants report low job demands and high job control). Job performance was entered as interval level data. Logistics regressions were first conducted to estimate the odds of moderate occupational sitting time and high occupational sitting time (against low occupational sitting time) due to demographic characteristics, health behaviours and work characteristics (job grade, working hours). Two regression models were constructed with occupational sitting as the dependent variable. In model 1, moderate sitting time was entered against the reference category of low sitting time. In model two, high sitting time was entered against the reference category of low sitting time. Two further multiple logistic regressions were then conducted to estimate the odds of moderate occupational sitting time (against low occupational sitting time) due to work engagement, job strain and job performance; and high occupational sitting time (against low occupational sitting time) and the same variables. The latter two models were adjusted first for age, BMI, smoking, physical activity, marital status, dependents and education; and then adjusted for the same covariates with the addition of working hours and job grade. Physical activity was included as a covariate to test whether the associations between the key variables and occupational sitting were independent of discretionary physical activity. Separate analyses were carried out for men and women. Data were analysed using SPSS statistics version 19.0 (SPSS Inc., Chicago, 2003).

## Results

Questionnaires were completed by 1945 men and 2491 women. Table 2 shows the characteristics of the study sample. Total occupational sitting time for the sample was an average of 379.63 minutes per day (s.d. 98.3) (an accumulated average of 6.32 hours per day at work). Mean occupational sitting time was lower for men (mean 362.4 minutes/day; s.d. 112.5) compared to women (385.7 minutes/day; s.d. 98.9) (t = 7.32; df =4434; p < .0001). Chi-Square analyses shows that the proportion of men classed as reporting low occupational sitting times was significantly greater than for women (35.9% versus 31.3%) and the proportion of women reporting high occupational sitting times was greater than for men (35.2% versus 31.0%; χ^2^ = 12.89, df = 2 *p* < 0.002).

**Table 2:** **Characteristics of study sample by gender**

**Characteristic**	**Men (n = 1945)**	**Women (n = 2491)**
**Mean occupational sitting (s.d)**	**362.4 (2.5)** **n (%)**	**385.7 (1.9)** **n (%)**
**Age**		
18-29 years	151 (7.8)	240 (9.6)
30-39 years	429 (22.1)	626 (25.1)
40-49 years	587 (30.2)	832 (33.4)
50-59 years	662 (34.0)	738 (29.6)
60-70 years	116 (6.0)	55 (2.2)
**BMI**		
Normal weight	557 (28.7)	1048 (42.2)
Overweight	940 (48.5)	834 (33.6)
Obese	442 (22.8)	603 (24.3)
Meets PA guidelines	464 (23.9)	426 (17.1)
Smoking (yes)	268 (13.8)	305 (12.3)
Married/cohabiting (yes)	1417 (72.9)	1674 (67.2)
Dependents (yes)	1170 (60.2)	1511 (60.7)
**Education**		
Up to general school certificate	269 (13.9)	643 (25.8)
A level (qualifications at end of college)	675 (34.8)	756 (30.3)
Degree level	403 (20.8)	477 (19.1)
Higher degree (e.g. MSc, PhD)	594 (30.6)	598 (24.0)
**Job grade**		
≥Senior civil servant/principal	213 (11.0)	176 (7.1)
Deputy principal	346 (17.8)	318 (12.8)
Staff officer	452 (23.2)	446 (17.9)
Executive officer	573 (29.5)	830 (33.3)
Administrative	315 (16.2)	690 (27.7)
Other	46 (2.4)	31 (1.2)
**Working hours**		
Full time (≥ 37.5 hours a week)	1857 (95.0)	1825 (73.3)
Part time (less than 37.5 hours a week)	98 (5.0)	666 (26.7)

Abbreviations: PA, physical activity. Definition of categories: PA guidelines = participating in at least 30 minutes of moderate-to-vigorous physical activity on ≥5 days; BMI categories: normal weight = BMI <25, overweight = BMI 25-29.9, obese = BMI ≥ -30. Part-time hours = less than 37.5 hours, full-time = 37.5 hours or more.

There were no significant differences in the proportion of men and women reporting work vigor. Proportionately more women than men reported high dedication (n = 1108, 44.5% versus n = 806, 41.4%; χ^2^ = 9.68, df = 2 *p* < 0.008); high absorption (n = 1099, 44.1% versus n = 643, 33.1%; χ^2^ = 53.91, df = 1 *p* < 0.0001); and work performance (n = 2054, 82.5% versus n = 1336, 68.7%; χ^2^ = 115.48, df = 1 *p* < 0.0001). There were no significant differences in the proportion of men and women reporting *low* job strain The proportion of men classed as experiencing *high* job strain was significantly greater when compared with women (n = 444, 22.8% versus n = 475, 19.1%; χ^2^ = 9.39, df = 1 *p* < 0.002).

Bivariate spearman correlations between key variables are shown in Table 3. The correlation coefficients are presented separately for men and women (women are presented in parenthesis). There are significant correlations between occupational sitting times and the three work engagement scales (for both genders) and job performance (men only) suggesting lower occupational sitting time is associated with better engagement and performance at work. There was no significant correlation between occupational sitting time and job control and job demands. Education was significantly correlated with the three engagement scales and with job grade suggesting that those with higher qualifications had higher work engagement and were in higher grade jobs (job grade was entered in descending order, hence the negative relationship). Job grade correlated with the three work engagement scales, job performance (except for men) job demands and job control. The three work engagement scales and job performance were positively correlated with job control. There was no relationship between vigor and job demands for either gender. Dedication, absorption and job performance were negatively correlated, albeit weakly, with job demands.

**Table 3:** **Spearman correlations between occupational sitting, physical activity levels, education, job grade, work engagement, job demands and performance**

	**M**	**SD**	**1**	**2**	**3**	**4**	**5**	**6**	**7**	**8**	**9**	**10**
1. Occupational sitting time (mean total minutes over five days)	362.44 (385.73)	112.54 (98.91)	-									
2. Physical activity (no. of days over 7 days)	2.83 (2.41)	2.10 (1.97)	-.10* (-.07*)	-								
3. Education level	3.68 (3.42)	1.05 (1.18)	03 (.04)	.03 (.04*)	-							
4. Job grade	4.32 (4.67)	1.36 (1.27)	-.05* (-.08*)	.01 (.01)	-.45* (.46**)	-						
5. Vigor (work engagement)	2.72 (2.69)	1.06 (1.04)	-.16** (-.07*)	.11* (.13**)	.12* (.07*)	-.20** (.14**)	-					
6. Dedication (work engagement)	3.23 (3.35)	1.19 (1.27)	-.12* (.05*)	.03 .06*)	.15** (.13**)	-.23** (.17**)	.74** (.68**)	-				
7. Absorption (work engagement)	3.45 (3.61)	0.63 (0.73)	-.08* (.07*)	-.01 (.02)	.15** (.13**)	-.17* (.18**)	.52** (.48**)	.62** (.64**)	-			
8. Job Demands	3.47 (3.53)	0.66 (0.72)	.01 (-.04)	.07* (.01)	-.16** (.19**)	-.05* (.32**)	.01 (.02)	-.05* (-.07*)	-.17** (-.17**)	-		
9. Job Control	3.59 (3.56)	0.63 (0.66)	.01 (.02)	.08* (.03)	.09* (.10*)	.14** (.17**)	.30** (.29**)	.33** (.32**)	.21** (.20**)	.28** (.31**)	-	
10. Job performance	5.39 (5.62)	1.07 (1.07)	-.11* (-.04)	.03 (.02)	-.03 (.09*)	.03 (.07*)	.32** (.27**)	.35** (.31**)	.24** (26**)	-.05* (-.07*)	14.**(.10*)	-

Note: Table presents spearman correlation coefficients for male and females. Coefficients for females are presented in parenthesis. *p < .05; **p < .01 All variables are on an interval or ordinal scale, ranging from low to high scores e.g. low mean sitting time to high mean sitting time); low job grade (other/Administrative) to high job grade (senior civil servant); low education (school certificate) to high education (higher degree).

Table 4 presents associations of demographic characteristics, health behaviours and work characteristics with occupational sitting using categories of low, moderate and high occupational sitting (with low occupational sitting used as a reference category against moderate and high sitting times) separately for men (n =1945) and women (n = 2491). Occupational sitting time was entered as the dependent variable with moderate sitting time and high sitting time entered against the reference category of low sitting time (data for low sitting is not presented in the table). The results for men show that the age groups ≥ 40 were less likely to be associated with high occupational sitting times. Males who met the physical activity guidelines were less likely to have high occupational sitting time. For women, those aged 40-49 years were less likely to have high occupational sitting time (compared to the reference group of those aged 18-29 years). For both genders, those who had dependents were less likely to report high occupational sitting times. There were no associations for BMI and educational level with occupational sitting times. For men, those in lower job grades were more likely to report lower occupational sitting times. For women, full-time workers were less likely to have high occupational sitting time.

**Table 4:** **Associations of demographic, health behaviours and work characteristics with occupational sitting categories of low, moderate and high occupational sitting (low occupational sitting used as reference category)**

	**Sitting time at work for men**				**Sitting time at work for women**			
	**Moderate sitting**		**High sitting**		**Moderate sitting**		**High sitting**	
	**OR**	**95% CI**	**OR**	**95% CI**	**OR**	**95% CI**	**OR**	**95% CI**
**Age**								
18-29 years	Reference		Reference		Reference		Reference	
30-39 years	1.04	(0.63 to 1.70)	0.84	(0.52 to 1.34)	1.02	(0.68 to 1.51)	0.90	(0.61 to 1.23)
40-49 years	0.83	(0.52 to 1.34)	**0.51**	**(0.33 to 0.82)**	**0.61**	**(0.42 to 0.90)**	**0.51**	**(0.35 to 0.74)**
50-59 years	0.78	(0.48 to 1.24)	**0.45**	**(0.29 to 0.72)**	0.81	(0.55 to 1.20)	0.70	(0.48 to 1.03)
60-70 years	0.77	(0.41 to 1.42)	**0.46**	**(0.24 to 0.84)**	1.02	(0.50 to 2.09)	0.45	(0.24 to 1.19)
**BMI**								
Normal weight	Reference		Reference		Reference		Reference	
Overweight	0.98	(0.76 to 1.27)	1.05	(0.80 to 1.37)	0.85	(0.68 to 1.07)	1.08	(0.86 to 1.35)
Obese	1.19	(0.88 to 1.63)	1.27	(0.92 to 1.75)	1.18	(0.91 to 1.52)	1.27	(0.98 to 1.66)
Meets PA guidelines	0.78	(0.61 to 1.00)	**0.64**	**(0.49 to 0.84)**	0.84	(0.65 to 1.09)	0.82	(0.63 to 1.07)
Smoking (yes)	0.96	(0.71 to 1.33)	0.67	(0.48 to 1.09)	0.97	(0.71 to 1.32)	0.97	(0.72 to 1.33)
Married/cohabiting (yes)	1.19	(0.89 to 1.60)	1.22	(0.91 to 1.64)	0.91	(0.72 to 1.12)	0.98	(0.76 to 1.24
Dependents (yes)	0.87	(0.67 to 1.14)	**0.72**	**(0.55 to 0.94)**	0.59	(0.47 to 0.76)	**0.48**	**(0.38 to 0.61)**
**Education**								
Up to school certificate	Reference		Reference		Reference		Reference	
(eg GCSE)								
A level	0.98	(0.67 to 1.38)	1.70	(0.16 to 17.71)	1.19	(0.39 to 3.66)	3.13	(0.76 to 12.82)
Degree level	1.19	(0.79 to 1.80)	2.20	(0.21 to 23.08)	1.12	(0.36 to 3.45)	2.99	(0.73 to 12.30)
Higher degree (e.g. MSc, PhD)	1.34	(0.86 to 2.01)	2.20	(0.21 to 22.92)	1.08	(0.34 to 3.25)	2.57	(0.62 to 10.76)
**Job grade**								
≥Senior Civil Servant /Principal	Reference		Reference		Reference		Reference	
Deputy Principal	1.09	(0.71 to 1.17)	0.85	(0.54 to 1.33)	1.16	(0.73 to 1.87)	1.44	(0.86 to 2.35)
Staff Officer	0.95	(0.62 to 1.15)	0.88	(0.57 to 1.36)	0.91	(0.58 to 1.43)	1.36	(0.86 to 2.17)
Executive Officer	**0.51**	**(0.33 to 0.78)**	**0.47**	**(0.30 to 0.71)**	0.67	(0.44 to 1.01)	0.71	(0.46 to 1.10)
Administrative	**0.59**	**(0.36 to 0.98)**	**0.43**	**(0.26 to 0.72)**	0.78	(0.50 to 1.21)	0.86	(0.56 to 1.37)
Other	**0.07**	**(0.02 to 0.24)**	**0.18**	**(0.08 to 0.42)**	**0.15**	**(0.04 to 0.56)**	0.63	(0.26 to 1.52)
Working full time	0.87	(0.51 to 1.50)	0.59	(0.32 to 1.10)	0.66	(0.52 to 8.38)	**0.52**	**(0.41 to 0.67)**

Statistical analysis: Logistic regression models with odds ratio (OR) and their 95% confidence interval. Results in bold indicate a significant finding. Data for reference category ‘low occupational sitting’ is not shown. PA = physical activity; working full time = 37.5 hours or more; reference category is ‘working part-time which is less than 37.5 hours’. Reference categories for physical activity is ‘not meeting guidelines of at least 30 minutes of moderate-to-vigorous physical activity on ≥5 days’; reference category for smoking is ‘not smoking’; reference category for married/cohabiting is ‘being single’, and reference category for dependents is ‘not having any dependents’.

Table 5 presents the associations of work engagement, job strain and job performance with occupational sitting categories of low, moderate and high occupational sitting (low occupational sitting used as reference category-data for low sitting is not presented in the table). For men, the unadjusted model in Table 5 shows those reporting good work vigor (average to high) were less likely to report high occupational sitting times (i.e., less likely to sit more than 420 minutes/day). In the first adjusted model, findings for high vigor and high dedication remained significant. In addition, those with high dedication were also less likely to report high occupational sitting time (as well as less likely to report moderate sitting time), indicating that high dedication is overall associated with low occupational sitting. Furthermore, those reporting low strain were less likely to sit for moderate lengths of time. In the fully adjusted model (adjusting for job grade and work pattern), except for low job strain, the associations for high vigor and high dedication with occupational sitting remained significant.

**Table 5:** **Associations of work engagement, job strain and job performance with occupational sitting categories of low, moderate and high occupational sitting (low occupational sitting used as reference category)**

	**Sitting time at work for men**				**Sitting time at work for women**			
	**Moderate sitting**	**High sitting**	**Moderate sitting**	**High sitting**				
	**OR**	**95% CI**	**OR**	**95% CI**	**OR**	**95% CI**	**OR**	**95% CI**
*Unadjusted*								
**Work engagement**								
*Vigor*								
Low	Reference		Reference		Reference		Reference	
Average	0.96	(0.69 to 1.34)	**0.71**	**(0.51 to 0.98)**	0.88	(0.66 to 1.17)	0.68	(0.51 to 0.90)
High	0.77	(0.55 to 1.07)	**0.50**	**(0.36 to 0.69)**	0.73	(0.56 to 0.98)	0.54	(0.40 to 0.72)
*Dedication*								
Low	Reference		Reference		Reference		Reference	
Average	0.90	(0.66 to 1.24)	0.87	(0.63 to 1.19)	1.09	(0.82 to 1.45)	1.24	(0.93 to 1.65)
High	**0.65**	**(0.46 to 0.92)**	0.70	(0.49 to 1.01)	0.94	(0.69 to 1.29)	1.21	(0.88 to 1.67)
*Absorption*								
Average	Reference	Reference	Reference	Reference				
High	1.11	(0.84 to 1.45)	1.14	(0.85 to 1.51)	1.02	(0.87 to 1.24)	**1.31**	**(1.04 to 1.67)**
High job strain (yes)	0.84	(0.63 to 1.12)	1.003	(0.74 to 1.37)	1.12	(0.85 to 1.49)	1.02	0.77 to 1.34)
Low job strain (yes)	0.73	(0.53 to 1.02)	1.04	(0.75 to 1.46)	1.01	(0.76 to 1.34)	0.84	(0.63 to 1.13)
Job performance	1.10	(0.89 to 1.40)	1.11	(0.87 to 1.43)	**1.37**	**(1.05 to 1.79)**	0.95	(0.74 to 1.23)
*Adjusted* ^*a*^								
**Work engagement**								
*Vigor*								
Low	Reference		Reference		Reference		Reference	
Average	0.98	(0.71 to 1.39)	0.74	(0.53 to 1.04)	0.85	(0.64 to 1.13)	**0.70**	**(0.52 to 0.93)**
High	0.81	(0.59 to 1.15)	**0.56**	**(0.40 to 0.79)**	0.75	(0.56 to 1.00)	**0.60**	**(0.44 to 0.80)**
*Dedication*								
Low	Reference	Reference	Reference	Reference				
Average	0.89	(0.65 to 1.23)	0.82	(0.64 to 1.22)	1.13	(0.85 to 1.50)	1.24	(0.92 to 1.67)
High	**0.64**	**(0.46 to 0.91)**	**0.69**	**(0.48 to 0.97)**	0.95	(0.70 to 1.30)	1.20	(0.86 to 1.66)
*Absorption*								
Average	Reference	Reference	Reference	Reference				
High	1.08	(0.83 to 1.43)	1.08	(0.80 to 1.44)	1.01	(0.80 to 1.28)	**1.29**	**(1.02 to 1.64)**
High job strain (yes)	0.84	(0.63 to 1.14)	1.08	(0.79 to 1.49)	1.11	(0.84 to 1.48)	1.02	(0.77 to 1.36)
Low job strain Yes)	**0.70**	**(0.50 to 0.98)**	0.92	(0.65 to 1.30)	0.95	(0.71 to 1.26)	0.82	(0.61 to 1.10)
Job performance	1.11	(0.87 to 1.42)	1.09	(0.85 to 1.41)	1.31	(0.95 to 1.72)	0.94	(0.72 to 1.21)
*Adjusted* ^*b*^								
**Work engagement**								
*Vigor*								
Low	Reference		Reference		Reference		Reference	
Average	0.90	(0.63 to 1.27)	0.72	(0.50 to 1.03)	0.89	(0.66 to 1.19)	**0.73**	**(0.54 to 0.96)**
High	0.70	(0.49 to 1.00)	**0.49**	**(0.34 to 0.70)**	0.79	(0.58 to 1.07)	**0.62**	**(0.45 to 0.84)**
*Dedication*								
Low	Reference	Reference	Reference	Reference				
Average	0.84	(0.61 to 1.18)	0.82	(0.59 to 1.14)	1.13	(0.84 to 1.51)	1.27	(0.94 to 1.72)
High	**0.65**	**(0.45 to 0.94)**	**0.68**	**(0.47 to 0.98)**	0.95	(0.68 to 1.31)	1.20	(0.86 to 1.68)
Absorption								
Average	Reference		Reference		Reference		Reference	
High	1.17	(0.88 to 1.55)	1.18	(0.82 to 1.39)	0.98	(0.76 to 1.25)	**1.29**	**(1.01 to 1.65)**
High job strain (yes)	0.84	(0.61 to 1.16)	1.03	(0.73 to 1.46)	1.10	(0.80 to 1.49)	0.91	(0.67 to 1.25)
Low job strain (yes)	0.75	(0.53 to 1.07)	0.96	(0.67 to 1.37)	1.01	(0.75 to 1.35)	0.84	(0.62 to 1.13)
Job performance	1.10	(0.85 to 1.42)	1.07	(0.82 to 1.40)	1.30	(0.98 to 1.73)	0.90	(0.69 to 1.17)

Statistical analysis: Logistic regression models with odds ratio (OR) and their 95% confidence interval. Results in bold indicate a significant finding. Data for reference category ‘low occupational sitting’ is not shown. For absorption, there were no participant data for the low absorption category. Therefore only two categories were used. Job performance is entered as interval data, where a high score indicates higher job performance. For high job strain and low job strain the reference category is ‘no’. ^a^Adjusted for age, BMI, physical activity, smoking, marital status, dependents and education; ^b^Further adjusted for job grade and working hours. *p* < .05.

For women, in the unadjusted model, those reporting higher levels of job performance were more likely to have moderate sitting times; and those with high absorption were more likely to report high sitting time. Those with good vigor (average to high) were less likely to report high occupational sitting time. After adjusting for the first set of covariates and the second set of covariates, job performance was not significant but the findings for high absorption and vigor remained significant.

## Discussion

This study examined the associations between occupational sitting and work engagement in a large cross-sectional sample of public sector office workers. Occupational sitting time was reported to be an average of 6.32 hours per workday. Overall, sitting times were lower for men than women, in contrast to that reported by Mummery et al in their Australian study [6] and Kazi et al [39] in their UK study of five organizational sectors including a Government sector, but reflect similar findings reported by Chau et al [40]. Total daily sitting time reported here are substantially higher than those reported in samples of working adults from non-UK studies [6,41], but are lower than the occupational sitting time reported by Kazi et al [39] who used the same occupational sitting time measure we used in this study.

In this study, data on interrupted sitting times were not collected. Studies that assessed how sedentary time is accumulated at work have reported that around 22-52% of occupational sitting is accrued in prolonged unbroken bouts (≥ 30 minutes) [42-44]. Considering that our sample of public sector office workers accumulated high volumes of workplace sedentary behaviour, it is possible that much of these sitting times were accrued in prolonged unbroken bouts. Such extended periods of uninterrupted sitting may have important health implications [45].

The study found that proportionately more women than men reported higher dedication, absorption and work performance. In contrast, men reported experiencing more job strain. The latter reflects the current literature [46] as men are likely to be in higher job grades and work longer hours. We also found that in comparison to women, men were in higher job grades and worked longer hours (Table 2). From previous discussions on work engagement it is assumed that work engagement is gender neutral [47]. However, our findings for two scales from the work engagement measure, dedication and absorption, add to the relatively small literature that this may not be the case and may indicate that women in the Irish public sector are more likely to report higher dedication and absorption in their work [47]. Further studies are required to examine the antecedents for the gender differences observed in our study.

Significant correlations were found for higher work engagement, job performance and job grade with lower occupational sitting times. These findings may suggest that employees who have higher work engagement are more likely to sit less; and/or that certain jobs or work tasks are structured in such a way that it is easier to either move away from the desk or to demonstrate work engagement and job performance. With regard to job tasks, support is partially provided by the logistic regression results that show lower job grades are associated with low occupational sitting times for men in this study. However, as this was a cross-sectional study in which causal relationships cannot be determined, interactions between work engagement, job performance and job grade with occupational sitting times need to be more formally explored using longitudinal data. The study found that the work engagement scales were positively correlated with job performance and job control, and negatively correlated with job demands (except for vigor, where no significant correlation was found). These results reflect previous research [24] that suggests that individuals are more likely to be engaged in their work if job control is high and demands are low [25].

No significant correlation was observed between educational status and occupational sitting times even though educational status showed significant correlations with job grade, work engagement and job performance. The logistic regression analysis also found no associations between educational status and occupational sitting for either men or women and is in contrast to previous studies [48-52].

Logistic regression analyses in this study found that men aged ≥40 years and women aged 40-49 years, were more likely to have low occupational sitting times. These findings could reflect differences in cultural norms across age groups which requires further exploration. Men who were meeting physical activity guidelines were more likely to have lower occupational sitting times. But these findings were not significant for women. Surprisingly there were no associations between BMI and occupational sitting as reported in other studies [40]. This is perhaps due to this study focusing solely on office workers or using self-report weight for calculating BMI and these results therefore, must be interpreted with caution.

Logistic regression results of work engagement, job strain and job performance with occupational sitting showed that when separating analyses for men and women, job performance was not associated with occupational sitting times. For work engagement, men were less likely to sit for prolonged periods at work (less than 421 minutes per day or less than 7 hours per day) if they reported experiencing high vigor (i.e. personal energy and resilience, willingness to invest effort). Furthermore, they were less likely to sit for prolonged periods at work (less that 360 minutes per day or 6 hours per day) if they reported high dedication (i.e. deriving a sense of significance from one’s work). These findings are independent of the influence of demographic characteristics, health behaviours, job grade and working hours.

The results for women showed that sitting for less than 421 minutes per day (or less than 7 hours per day) at work was associated with average-high levels of vigor irrespective of demographic characteristics, health behaviours, job grade and working hours. In contrast, high levels of absorption were associated with an increase in the odds of high occupational sitting times. As Schaufeli and Bakker [23] posit that absorption resembles being immersed in one’s work and having difficulties in detaching oneself from it, our findings may suggest that women who are absorbed with their work are less likely to take a break, stand up and/or move around [22,23]. Further research is required to understand the mechanism of this relationship. Nevertheless, the current findings suggests that there is much work to be done on decreasing occupational sitting time among women who are absorbed in their work, without disrupting their concentration or flow.

Overall, the present findings suggest that after controlling for confounding variables in separate logistic regression models for men and women, high levels of vigor (and dedication for men) are associated with low levels of occupational sitting. Studies examining work engagement clearly show the benefits of a workforce that is engaged such as being more productive and having good mental and physical health [22,23,26]. Our findings contribute to this literature in that employees with high work engagement are less likely to have prolonged sitting times at work. Our findings also add to the growing evidence highlighting the workplace as an important setting for reducing sedentary behaviour [51]. Our study results may provide an incentive for organizations to reduce or interrupt sitting times at work. However, as this is a cross-sectional study, further longitudinal studies are required to understand the interactions between high work engagement and low occupational sitting time, but also between job strain and occupational sitting as our findings were not consistent or conclusive for this.

This study has a number of strengths. First, the study has a large sample size of office-based workers with varying educational levels and job grades allowing for the generalization of findings. Second, all self-report measures used in this study are validated and reliable. The organizational measures used here are considered to be important variables that capture employee health and performance and have been widely used in previous research. Third, our study contributes to the literature on workplace sedentary behaviour and work engagement and is one of the first studies to examine the relationship between sedentary behaviour and work engagement.

This study also has a number of limitations. First, the study adopted a cross-sectional design, limiting the extent to which reverse causation and alternate pathways between occupational sedentary behaviour and measures of organizational health could be tested. This study, however, reports findings from the first wave of survey data collected from NICS. A second survey will be distributed shortly and prospective analyses are planned. Second, all measures used in this study are self-report measures which may be prone to recall bias and under-reporting or over estimating work performance and sedentary behaviour. This is more of an issue for reporting on sedentary behaviour as this can be measured objectively whereas it is more difficult to objectively measure multi-dimensional aspects of individual work performance and health. The sitting-time questionnaire used in this study however, has been reported to be comparable to total sedentary time measured via accelerometry [21]. Third, whilst the study has a large sample, the poor response rate (20%) may be considered as a limitation. Nevertheless response rates between 20-25% are common in workplace organisational and wellbeing studies such as this [52-54]. Finally, actual working time including overtime hours was not collected. As workers may vary in their actual hours worked, this might influence work engagement. Therefore, our findings on work engagement must be interpreted with caution.

## Conclusions

Overall, this study showed that being actively engaged in one’s work is associated with lower occupational sitting times for men (vigor and dedication) and to a limited extent for women also (vigor only). This suggests that interventions such as introducing sit-stand workstations to reduce sitting times, may be beneficial for men’s working experience but may not be for women. Longitudinal studies are planned to further test these findings.
